# Effect of dapagliflozin on the prognosis of patients with acute myocardial infarction undergoing percutaneous coronary intervention

**DOI:** 10.1186/s12933-022-01627-0

**Published:** 2022-09-16

**Authors:** Yi Zhu, Jia-li Zhang, Xue-jiao Yan, Ling Sun, Yuan Ji, Fang-fang Wang

**Affiliations:** 1 Department of Cardiology, The Affiliated Changzhou Second People’s Hospital of Nanjing Medical University, Changzhou, 213000 People’s Republic of China; 2 Department of Gastroenterology Centre, The Affiliated Changzhou Second People’s Hospital of Nanjing Medical University, Changzhou, 213000 People’s Republic of China

**Keywords:** DAPA, AMI, MACE, TyG, AIP

## Abstract

**Background and aims:**

The effect of dapagliflozin (DAPA) on the prognosis of patients with acute myocardial infarction (AMI) is unclear. The present study was conducted to evaluate the association between DAPA administration and adverse events in patients with AMI undergoing percutaneous coronary intervention (PCI).

**Methods:**

This single-center retrospective analysis study included a total of 786 patients with AMI from January 2019 to August 2021 who were or were not administered DAPA at discharge. The primary endpoint was the composite of major adverse cardiovascular events (MACE), including overall deaths, heart failure, nonfatal MI, nonfatal stroke, and unplanned repeat revascularization (URR). Differences in the triglyceride glucose (TyG) index and the atherogenic index of plasma (AIP) both during hospitalization and 12 months after discharge (if achievable) were also compared.

**Results:**

During a median follow-up of 23 months, 130 patients had MACE (118 in the DAPA-free group and 12 in the DAPA group). Kaplan–Meier survival analyses revealed that the cumulative incidence of MACE (log-rank test, p = 0.009), heart failure (p = 0.003), nonfatal MI (p = 0.005), and URR (p = 0.031) was higher in the DAPA-free group. In addition, the multivariate Cox analysis showed that DAPA was significantly associated with the reduced risk of MACE (hazard ratio = 0.170, 95% confidence interval = 0.078–0.373, p < 0.001). Considering each specific adverse event, the DAPA-free group was associated with heart failure, nonfatal MI, and URR in multivariate Cox regression analyses. Stratification analyses suggested that DAPA has a strong protective effect in patients with AMI of advanced age with concomitant diabetes or those who are not on angiotensin receptor enkephalinase inhibitors. Furthermore, the TyG index and AIP of the patients 12 months after DAPA administration at discharge were significantly lower than those during hospitalization.

**Conclusions:**

DAPA is an independent protective factor against MACE and may provide incremental prognostic information in patients with AMI undergoing PCI.

## Introduction

Despite extensive progress in the field of interventional therapy, the incidence of major adverse cardiovascular events (MACE) in patients with acute myocardial infarction (AMI) is still high. Risk factors such as diabetes, chronic kidney disease (CKD), atrial fibrillation, high coronary disease burden, low ejection fraction, and advanced age may worsen the prognosis of such patients [1–3]. SGLT2 inhibitors (SGLT2i) have been shown to improve cardiorenal endpoints in patients with type 2 diabetes (T2DM), CKD, and chronic heart failure with reduced ejection fraction (HFrEF) [4–6]. The EMPA-REG OUTCOME trial showed that SGLT2i improved the admission rate of cardiovascular mortality and HF in patients with a long-term history of MI [7, 8]. However, it is not clear whether SGLT2i can improve outcomes in patients with AMI.

Considering the residual risk under current treatment strategies and the growing clinical evidence that support the use of SGLT2i, patients with a long term coronary artery disease are more likely to suffer from coexisting SGLT2i indications (such as T2DM or HF). Therefore, it is difficult to directly determine whether the benefit of SGLT2i observed in patients with T2DM or HF may also benefit those with concomitant AMI. Despite the contiguous spectrum of treatments, trials in those with AMI are needed to validate consistent treatment effects. At present, there is a large gap in evidence regarding SGLT2i in the HF risk spectrum of patients with AMI. Previous studies on the benefits of SGLT2i in patients with HF have primarily focused on HF stages A/C/D; however, fewer studies have evaluated HF stage B patients with AMI, i.e., the presence of cardiac structural changes without any clinical signs of HF [9]. However, this population constitutes a considerable proportion of patients with HF. Unfortunately, in previous large-scale studies (EMPA-REG OUTCOME (BI 10,773 [Empagliflozin] Cardiovascular Outcome Event Trial in Type 2 Diabetes Mellitus Patients), CANVAS (CANagliflozin cardioVascular Assessment Study), CREDENCE (Computed Tomographic Evaluation of Atherosclerotic Determinants of Myocardial Ischemia), and others), these patients were completely excluded at the time of enrollment [7, 10, 11]. Thus, there are several questions regarding the therapeutic efficacy of SGLT2i in patients with AMI that need to be answered via clinical studies.

In the present study, the association between dapagliflozin (DAPA), an SGLT2i, and MACE in patients with AMI undergoing percutaneous coronary intervention (PCI) was explored. In addition, the effects of DAPA on coronary artery disease-related indicators were evaluated.

## Patients and methods

### Ethics approval

This study was approved by the Committee of Clinical Investigation of The Affiliated Changzhou No.2 People’s Hospital of Nanjing Medical University (2020-KY253-01) and conducted in accordance with the guidelines of the Declaration of Helsinki.

### Participants

This was a single-center, retrospective analysis derived from a prospective observational study (ChiCTR1800014583) on patients with AMI undergoing PCI that was conducted between January 2019 and August 2021 at The Affiliated Changzhou No.2 People’s Hospital of Nanjing Medical University. All the enrolled patients have not used SGLT2i drugs before. For this study, patients were consecutively enrolled and followed-up 1 month after discharge and every 3 months thereafter. The general condition, vital signs, hospitalization and medication status were consulted at each follow-up visit, and patients were invited to the outpatient clinic if necessary.

**Fig. 1:**
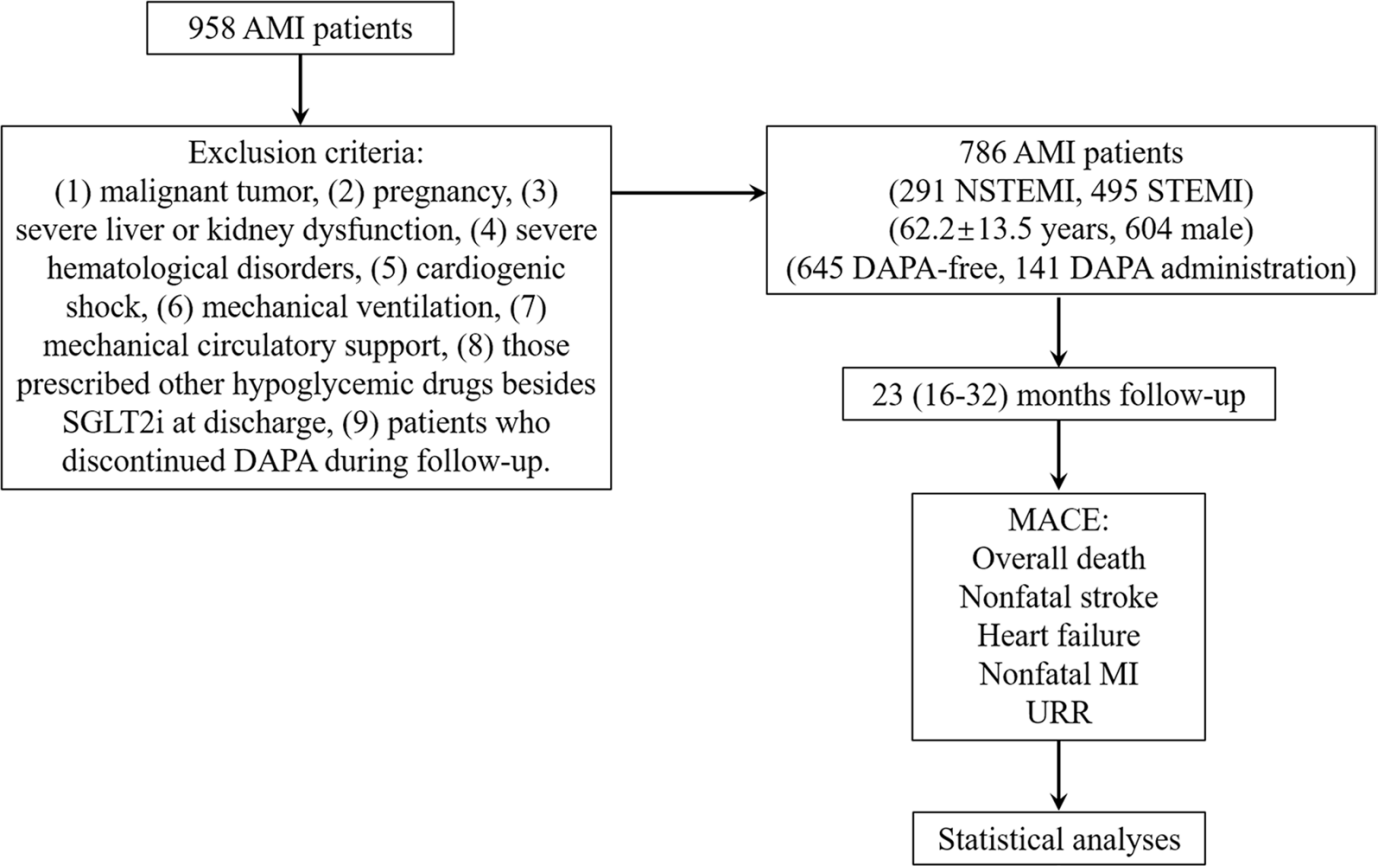
Study flow. Patients selection, exclusion criteria, follow-up and the definition of MACE

The exclusion criteria of this study were as follows: patients with malignant tumor, pregnancy, severe liver or kidney dysfunction, severe hematological disorders, cardiogenic shock, mechanical ventilation, mechanical circulatory support, those prescribed other hypoglycemic drugs besides SGLT2i at discharge and patients who discontinued DAPA during follow-up. A considerable number of DM patients were excluded, and most of the selected DM patients were in the early stage of DM, mainly relying on diet control and exercise. Severe liver dysfunction was considered as elevated serum transaminases, severely elevated serum bilirubin, decreased albumin concentration and coagulation disorders. Severe kidney dysfunction was considered as CKD stage IV or V. Severe hematological disorders were defined as multiple myeloma, lymphoma, myelodysplastic syndrome or leukemia (Fig. 1).

### Data retrieval

Demographic information, clinical characteristics, clinical events, medical histories, treatment, laboratory examinations, and medical instrument inspection records during hospitalization were retrieved from the hospital’s electronic medical record system.

In this study, arrhythmia (within 1 week of admission) was defined as at least one episode of atrial fibrillation, atrial flutter, ventricular fibrillation, or ventricular flutter. Hypertension was considered as a systolic blood pressure of > 140 mmHg and/or diastolic blood pressure (DBP) of > 90 mmHg and/or receiving antihypertension treatment. A fasting plasma glucose of > 7.0 mmol/L, casual plasma glucose of > 11.1 mmol/L, 2-h blood glucose of > 11.1 mmol/L after an oral glucose tolerance test, and/or use of antidiabetic medicine were considered to indicate diabetes.

The Gensini score was used to assess the severity of coronary artery disease and calculated as shown previously [12]. The atherogenic index of plasma (AIP) was defined as the logarithm to the base 10 of the ratio of fasting plasma triglyceride (TG) (mg/dL) to high-density lipoprotein cholesterol (HDL-C) [log (TG/HDL-C)], and the TG glucose (TyG) index was calculated as Ln (fasting TG (mg/dL) × fasting blood glucose (mg/dL) / 2).

Information on MACE was obtained via telephone calls or from the medical records system. The primary endpoint was the composite of MACE, including overall deaths, HF, nonfatal MI, nonfatal stroke, and unplanned repeat revascularization (URR). HF was diagnosed as per the typical symptoms, signs, and laboratory tests, including orthopnea, acute pulmonary edema, and brain natriuretic peptide (BNP) testing. MI was considered as [1] symptoms of ischemic chest pain; [2] a characteristic ST-T dynamic evolution on the electrocardiogram, or with abnormal q waves; [3] serum myocardial enzyme spectrum increased and decreased. Nonfatal MI was diagnosed as new pathological Q waves in ≥ 2 contiguous electrocardiogram leads according to the previous study [13]. Patients with an acute ischemic cerebral vascular event were diagnosed with stroke, and any nonstaged revascularization after PCI was considered URR (Fig. 1).

### Statistical analyses

All data were subjected to a normal distribution test and are expressed as the mean ± standard deviation for approximately normally distributed data and as the median (interquartile range) for skewed continuous data. Continuous variables between two groups were compared using Student’s t-test or the Mann–Whitney U-test according to their distribution. The chi-squared test was used for comparing categorical variables between the groups. Cumulative incidence curves were visualized using the Kaplan–Meier analyses, and the log-rank test was performed. Cox proportional hazard analyses with six models were performed to detect any independent risk factors by computing the hazard ratio with a 95% confidence interval (CI). Subgroup analyses according to the factors of age, sex, hypertension, diabetes, low-density lipoprotein cholesterol (LDL-C), and angiotensin receptor enkephalinase inhibitor (ARNI) administration were performed. All tests were two tailed, and p values < 0.05 were considered significant. All statistical analyses were performed using SPSS software 22.0 (SPSS Inc., Chicago, IL, USA).

## Results

A total of 786 patients with a median follow-up of 23 months (interquartile range: 16–32 months) were included in the study; their mean age was 62 ± 14 years and 604 (76.8%) patients were men. The baseline characteristics of the participants are summarized in Table 1. Higher proportion of patients who received DAPA had hypertension or diabetes. These patients also had higher BNP and HbA1c levels as well as larger left ventricular end-diastolic diameters but lower HDL-C, creatine phosphokinase, and left ventricular ejection fraction levels. In angiography, patients with DAPA intervention had a higher Gensini score. With regard to drugs, higher proportion of patients who received DAPA also received ARNI and mineralocorticoid receptor antagonist at discharge.

**Table 1:** Baseline characteristics of the study population

Characteristics	DAPA-free (n = 645)	DAPA (n = 141)	P value
Age (years)	62.5 ± 13.5	60.6 ± 13.6	0.894
Sex, male, n (%)	497 (77.1)	105 (74.5)	0.452
BMI (kg/m2)	24.4 ± 3.9	26.2 ± 4.1	0.885
Smoking, n (%)	319 (49.5)	63 (44.7)	0.281
Hypertension, n (%)	393 (60.9)	104 (73.8)	0.005
Diabetes, n (%)	96 (14.9)	96 (68.1)	< 0.001
Cerebral infarction, n (%)	40 (6.2)	8 (5.7)	0.867
In-hospital arhythmia, n (%)	47 (7.3)	12 (8.5)	0.628
In-hospital AHF, n (%)	57 (8.8)	14 (9.9)	0.694
Length of stay (days)	8 (6.8–9)	8 (7–10)	0.220
STEMI, n (%)	396 (61.4)	99 (70.2)	0.050
Stenting/POBA, n (%)	627 (97.2)	136 (96.5)	0.398
Multivessel disease, n (%)	616 (95.5)	130 (92.2)	0.106
Gensini score	42 (21–69)	49 (34–80)	0.002
Biochemical test			
ALP (U/L)	76.5 ± 25.6	77.9 ± 20.5	0.658
UA (umol/L)	331 (280–400)	324 (246–393)	0.112
LDL-C (mmol/L)	2.58 ± 0.88	2.77 ± 0.89	0.946
HDL-C (mmol/L)	1.06 ± 0.30	1.01 ± 0.26	0.023
TC (mmol/L)	4.3 ± 1.1	4.6 ± 1.4	0.677
TG (mmol/L)	1.42 (1.05–1.99)	1.76 (1.23–2.42)	0.132
BNP (pg/mL)	330 (99–1120)	521 (145–1985)	< 0.001
CPK (U/L)	534 (133–1446)	486 (185–1276)	0.028
CK-MB (U/L)	51.4 (21.2–123.1)	52.4 (21.9–109.9)	0.055
HBDH (U/L)	351 (196–672)	337 (198–571)	0.338
HbA1c (%)	6.2 ± 1.2	7.9 ± 1.7	< 0.001
Ccr (ml/min)	66 ± 43	69 ± 33	0.917
Ultrasonic cardiogram			
LA (mm)	3.9 ± 0.5	4.0 ± 0.5	0.553
LV (mm)	5.1 ± 0.5	5.3 ± 0.6	0.015
EF (%)	53 ± 9	49 ± 10	0.021
Pharmacological intervention			
β-block, n (%)	364 (56.4)	81 (57.4)	0.817
ACEI/ARB, n (%)	213 (33.0)	39 (27.7)	0.218
ARNI, n (%)	126 (19.5)	42 (29.8)	0.007
MRA, n (%)	50 (7.8)	16 (11.3)	< 0.001

Values are shown as the means ± SD, median (interquartile range) or percentage

*BMI* body mass index, *POBA* plain old balloon angioplasty, *ALP* alkaline phosphatase, *UA* uric acid, *LDL-C* low-density lipoprotein cholesterol, *HDL-C* high-density lipoprotein cholesterol, *TC* total cholesterol, *TG* triglycerides, *BNP* brain natriuretic peptide, *CPK* creatine phosphokinase, *CK-MB *creatine kinase-MB, *HDBH* hydroxybutyrate dehydrogenase, *Ccr* creatinine clearance rate, *LA* left atrium, *LV* left ventricle, *EF* ejection factor, *ARNI* angiotensin receptor-neprilysin inhibitor, *MRA* mineralocorticoid receptor antagonist

**Fig. 2:**
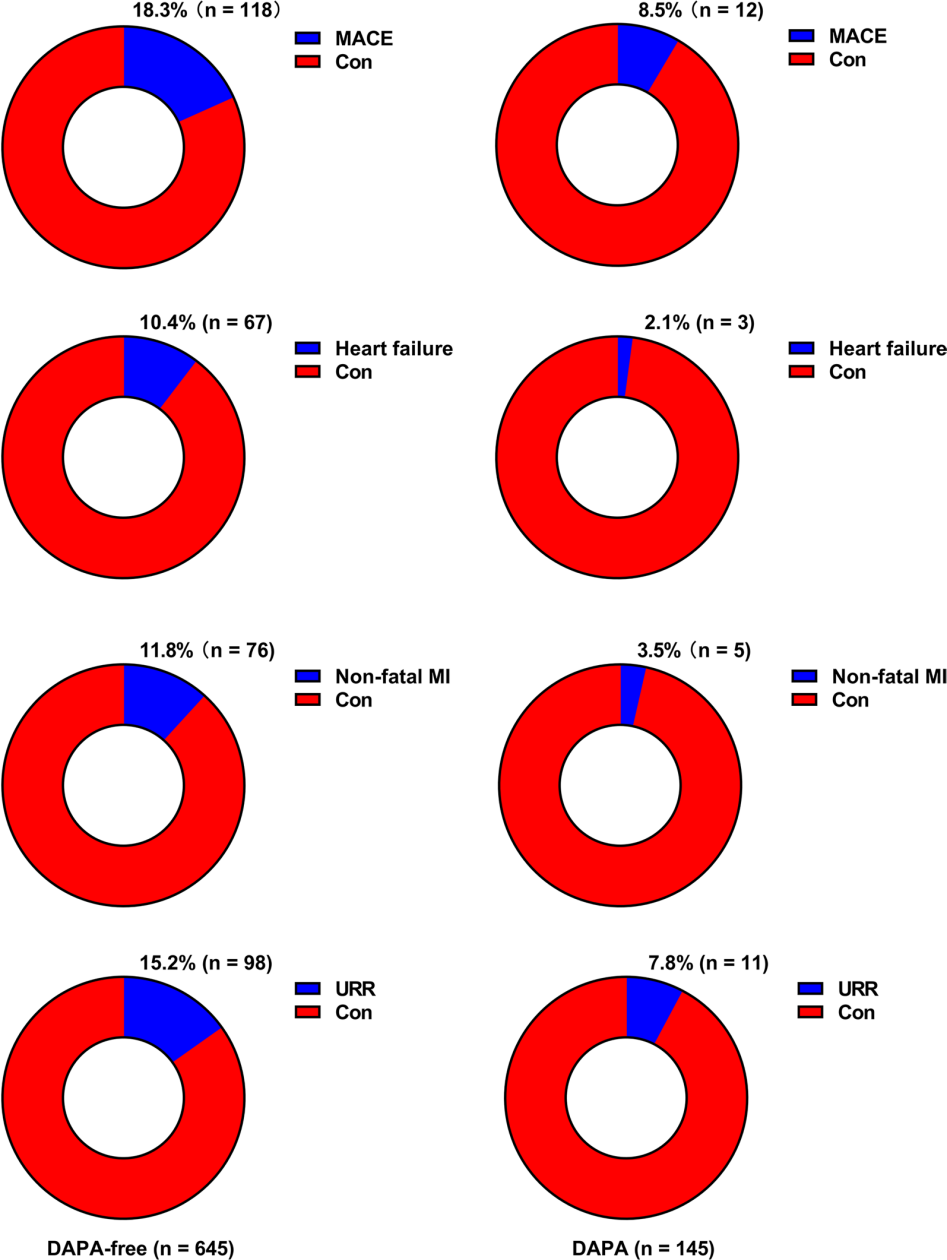
Presentation of adverse cardiovascular events during follow-up

**Fig. 3:**
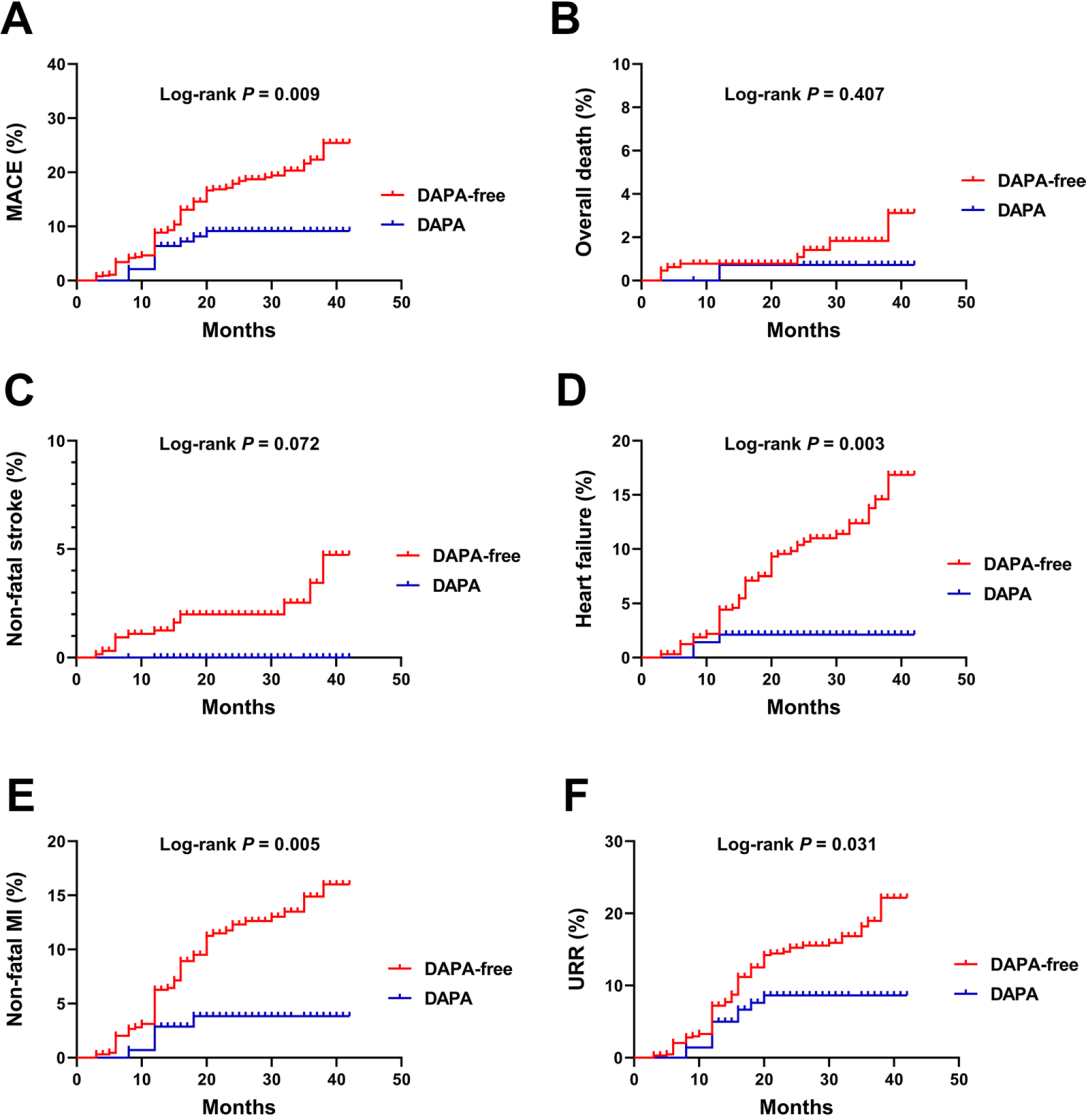
Cumulative incidence plots of MACE and each component of MACE stratified by the DAPA administration. **A** MACE; **B** overall death; **C** non-fatal stroke; **D** heart failure; **E** non-fatal MI; **F** URR. MACE: major adverse cardiovascular events; MI: myocardial infarction; URR: unplanned repeat revascularization.

At follow-up, more patients without DAPA administration had MACE. Moreover, a higher percentage of these patients had HF, nonfatal MI, and URR (all p < 0.05) (Table 2 and Fig. 2). Kaplan–Meier survival analysis revealed that the cumulative incidence of MACE increased without DAPA therapy (log-rank test, p = 0.009) (Fig. 3). Considering each component of MACE, patients without DAPA administration at discharge had a higher cumulative incidence of HF (p = 0.003), nonfatal MI (p = 0.005), and URR (p = 0.031) (Fig. 3).

**Table 2:** Adverse cardiovascular events according to the DAPA administration during follow-up

Adverse Cardiovascular events	DAPA-free	DAPA	P value
	n = 645	n = 141	
MACE, n (%)	118 (18.3)	12 (8.5)	0.018
Overall death, n (%)	9 (1.4)	1 (0.7)	0.440
Heart failure, n (%)	67 (10.4)	3 (2.1)	< 0.001
Non-fatal MI, n (%)	76 (11.8)	5 (3.5)	0.001
Non-fatal stroke, n (%)	15 (2.3)	0 (0)	0.050
URR, n (%)	98 (15.2)	11 (7.8)	0.012

The MACE was defined as the composite of overall death, HF, non-fatal MI, non-fatal stroke, and URR

*MACE* major adverse cardiovascular events; *MI* myocardial infarction; *URR* unplanned repeat revascularization

DAPA administration at discharge was significantly associated with a lower risk of MACE in both univariate and multivariate Cox regression analyses (p < 0.05 for all models) (Table 3). Furthermore, considering each specific adverse event, DAPA administration was associated with the reduced risk of HF, nonfatal MI, and URR in both univariate and multivariate Cox regression analyses (Table 4).

**Table 3:** Univariate and multivariate Cox proportional hazard analyses for the MACE according to the DAPA administration

		HR	95% CI	P value
Model 1	DAPA-free	Reference		
	DAPA	0.470	0.259–0.851	0.013
Model 2	DAPA-free	Reference		
	DAPA	0.480	0.265–0.871	0.016
Model 3	DAPA-free	Reference		
	DAPA	0.440	0.233–0.833	0.012
Model 4	DAPA-free	Reference		
	DAPA	0.426	0.225–0.808	0.009
Model 5	DAPA-free	Reference		
	DAPA	0.422	0.223–0.800	0.008
Model 6	DAPA-free	Reference		
	DAPA	0.391	0.193–0.792	0.009
Model 7	DAPA-free	Reference		
	DAPA	0.388	0.192–0.785	0.008

Model 1: Unadjusted

Model 2: Adjusted for age, sex

Model 3: Model 2 + smoking, hypertension, diabetes, cerebral infarction

Model 4: Model 3 + STEMI/NSTEMI + PCI details (stenting/POBA, multivessel disease or not)

Model 5: Model 4 + in-hospital AHF, in-hospital arhythmia

Model 6: Model 5 + HDL-C, LDL-C, TC, TG, AIP, TyG, CPK, CK-MB, HBDH, BNP, HbA1c

Model 7: Model 6 + β-block at discharge, ACEI/ARB at discharge, ARNI at discharge, MRA at discharge

**Table 4:** Univariate and multivariate Cox proportional hazard analyses for adverse prognosis according to the DAPA administration

		Univariate analyses				Multivariate analyses		
		HR	95% CI	P value		HR	95% CI	P value
Heart failure	DAPA-free	Reference				Reference		
	DAPA	0.206	0.065–0.655	0.007		0.114	0.030–0.439	0.002
Non-fatal MI	DAPA-free	Reference				Reference		
	DAPA	0.301	0.122–0.743	0.009		0.129	0.043–0.387	< 0.001
URR	DAPA-free	Reference				Reference		
	DAPA	0.514	0.276–0.959	0.036		0.172	0.075–0.394	< 0.001

Multivariate analyses were based on model 7 in Table 3

*MI* myocardial infarction, *URR* unplanned repeat revascularization

**Fig. 4:**
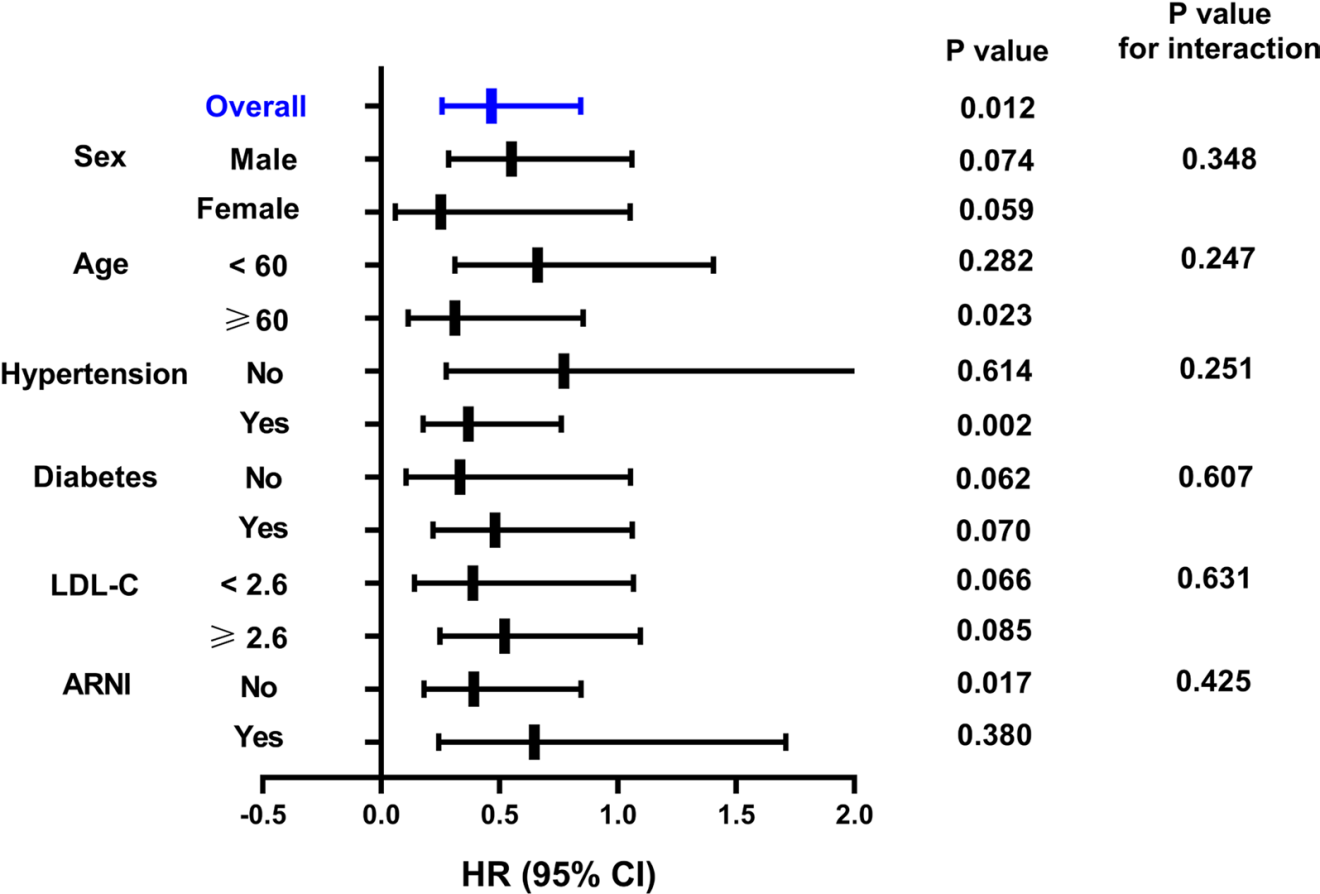
Subgroup analyses of DAPA for MACE

As shown in Fig. 4, when stratified by age, the occurrence rate of MACE in patients aged > 60 years in the DAPA group was 0.312-fold lower than that in the DAPA-free group (95% CI, 0.114–0.854, p = 0.023). In patients with hypertension, the risk of MACE in the DAPA group was 0.369-fold lower than that in the DAPA-free group (95% CI, 0.178–0.362, p = 0.002). The predictive values in patients without hypertension were not statistically significant. For patients without ARNI at discharge, the risk of MACE in the DAPA group was 0.392-fold lower than that in the DAPA-free group (95% CI, 0.182–0.845, p = 0.017). However, in the subgroup that received ARNI treatment, no statistically significant difference was observed with respect to the use of DAPA for predicting MACE. Besides, when stratified by sex, diabetes, or LDL-C levels, no significant differences in MACE were found between the DAPA and DAPA-free groups.

**Fig. 5:**
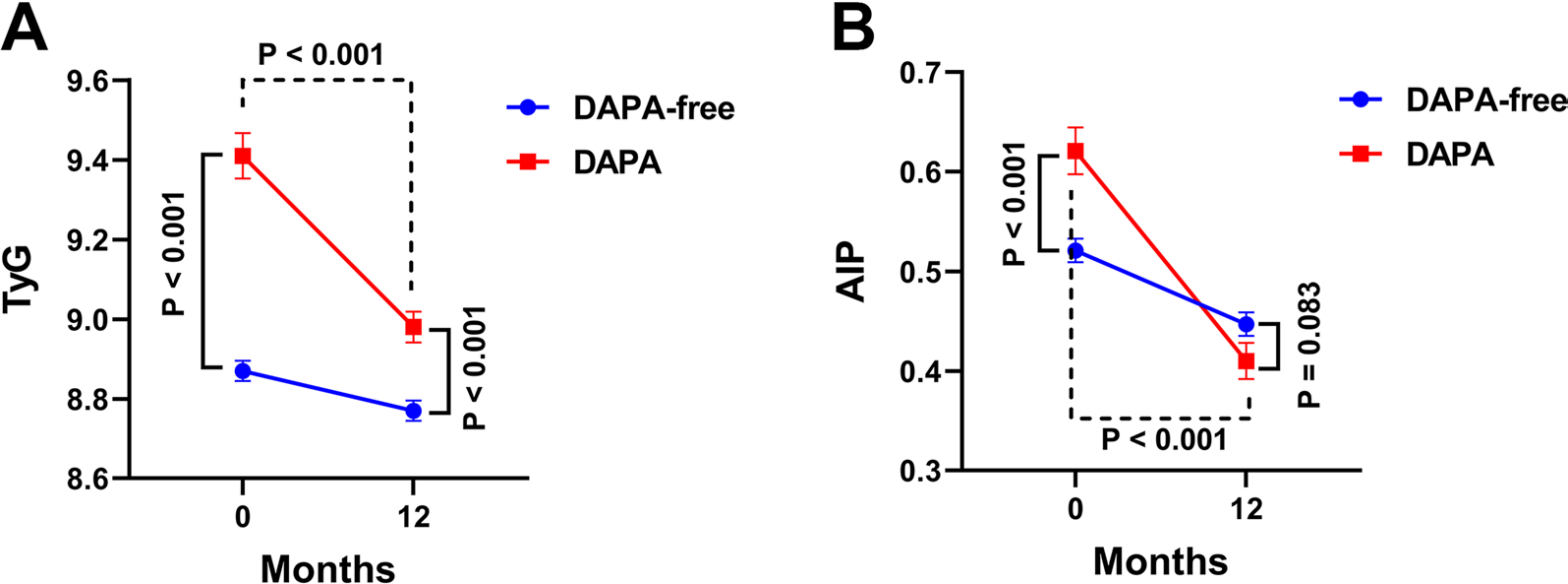
Effects of DAPA on TyG and AIP index. TyG (**A**) and AIP (**B**) index were calculated in hospital and 12 months post-discharge

As AIP and the TyG index are associated with MACE in patients with AMI undergoing PCI [14, 15], the effects of DAPA administration on AIP and the TyG index 12 months after discharge were evaluated. As shown in Fig. 5, AIP and the TyG index in the DAPA group were significantly higher than those in the hospitalized DAPA-free group. After 12 months, AIP and the TyG index were significantly decreased with DAPA administration, particularly due to the effect of DAPA on AIP.

## Discussion

The present study was conducted to evaluate the association between DAPA intervention and MACE in patients with AMI undergoing PCI. The results showed that DAPA administration was associated with reduced MACE in patients with AMI, particularly in patients with advanced age and hypertension and in those who did not receive ARNI. Moreover, the study revealed that DAPA administration significantly decreased AIP and the TyG index 12 months after discharge.

Current treatment guidelines and expert consensus generally state that SGLT2i should be avoided whenever possible during acute progression, including in acute MI, to avoid hypovolemia, hypotension, ketoacidosis, or acute kidney injury. However, in clinical trials on acute and chronic HF, the risk of these adverse events in the SGLT2i treatment group was uncommon and not significantly higher than that in the placebo group [6, 16]. Another safety concern during the perioperative management of patients with AMI is the repeated use of angiographic contrast agents during PCI, which may cause contrast-associated acute kidney injury. Considering the effect of SGLT2i on the afferent arteriole tone, they may cause a small reduction in the glomerular filtration rate. However, there is sufficient evidence suggesting that SGLT2i stabilize long-term renal function in patients with T2DM, CKD, and HFrEF, thereby providing a rationale for their safe use in patients with underlying renal impairment during AMI [17, 18]. Based on the yet unclear relationship between SGLT2i and the prognosis of patients with AMI, 786 patients with AMI admitted for PCI in a single center who were followed-up after discharge were retrospective analyzed. The analysis revealed that the use of DAPA is biased toward patients with T2DM; however, early intervention with SGLT2i is still preferred for some non-T2DM patients with AMI in the acute phase. In addition, the study revealed that the use of DAPA is biased toward patients with more adverse cardiovascular factors, which to a certain extent suggests that patients with AMI with more underlying diseases are more suitable for early intervention with SGLT2i.

In the median follow-up of 23 months (follow-up is being continued), the reported cases of overall deaths and nonfatal stroke were few; thus, there was a lack of statistical significance between the DAPA and DAPA-free groups. Therefore, in the Cox subgroup analyses, overall deaths and nonfatal stroke were not analyzed separately. Kaplan–Meier survival and Cox regression analyses indicated that early intervention with DAPA could reduce the occurrence of total MACE events, HF, nonfatal MI, and URR. Studies have shown that advanced age and hypertension are highly correlated with MACE [19] and that early intervention with ARNI has a protective effect on the prognosis of patients with AMI, particularly in the prognosis of patients with AMI complicated with HFrEF [20]. In the present study, subgroup analysis further suggested that the early intervention of DAPA significantly reduces MACE events in patients with advanced age and hypertension or those who did not receive ARNI. Because this was a single-center study, the number of included cases is small and the retrospective analysis cannot avoid the interference of exposure factors. However, the study results provide some idea about the relationship between the early intervention of SGLT2i and prognosis of patients with AMI.

It has been shown that dapagliflozin improves micro- and macrovascular endothelial function compared to glibenclamide, regardless of glycemic control in patients with T2DM and subclinical carotid atherosclerotic disease [21] and ameliorates angiotensin II-induced cardiac remodeling by regulating the transforming growth factor-beta1/Smad signaling in a non-glucose-lowering dependent manner [22]. Moreover, dapagliflozin induces vasodilation in resistance-size mesenteric arteries by stimulating smooth muscle cell K_V_7 ion channels [23] and attenuates advanced glycation end product-induced inflammation and apoptosis through activating AMP-activated protein kinase- mammalian target of rapamycin mediated autophagy pathway in podocytes [24]. The mechanisms of the efficacy of SGLT2i in AMI include the attenuation of neurohormonal activation, cardiomyocyte necrosis, and reperfusion injury. In addition, coronary blood flow and ventricular load are improved by enhancing the endothelial function and vasodilation, thereby improving myocardial energy metabolism as well as contractility and other functions. The ultimate effect of SGLT2i is the reversal of cardiac enlargement, abnormal rhythm, and myocardial fibrosis and the improvement of heart failure. In addition to the heart, it can indirectly improve the cardiorenal axis and improve the prognosis of patients by decreasing the intraglomerular pressure and increasing erythropoietin production [25–28]. Considering that glucose and lipid metabolisms are closely associated with endothelial phenotype stability, myocardial fibrosis, and myocardial cell necrosis, changes in the TyG index and AIP, the two indicators related to glucose and lipid metabolisms, were tracked to reveal the possible scope of SGLT2i action. The results revealed that the use of DAPA at discharge significantly reduced the TyG index and AIP for up to 12 months. Although the two indices also decreased in the DAPA-free group, this may have occurred because of the action of statin. It is not difficult to infer from results shown in Fig. 3 that the effect of DAPA combined with statin is significantly better than that of statin alone. This suggests that the target of DAPA may not be limited to glucose metabolism but may also have regulatory effects on lipid metabolism. A recent study has shown that SGLT2i has no significant effect on plasma PCSK9 levels, suggesting that it is safe for a possible combination therapy in the future [29].

## Limitations

First, this was a single center study based on the Chinese population, and DAPA was not validated in other populations. Second, long-term evaluation was not conducted. Third, the predictive value of DAPA based on the factors measured at discharge could not be determined. Finally, we did not perform propensity score matching due to the small sample size included.

## Conclusions

DAPA was a reliable and independent protective factor against MACE in patients with AMI undergoing PCI.

## Data Availability

The datasets used and/or analyzed during the current study are available from the corresponding author on reasonable request.
